# Current challenges and therapeutic advances of CAR-T cell therapy for solid tumors

**DOI:** 10.1186/s12935-024-03315-3

**Published:** 2024-04-15

**Authors:** Tong Chen, Mingzhao Wang, Yanchao Chen, Yutao Liu

**Affiliations:** Department of Medical Oncology, National Cancer Center/National Clinical Research Center for Cancer/Cancer Hospital, Chinese Academy of Medical Sciences & Peking Union Medical College, No. 17 Panjiayuan Nanli, Chaoyang District, Beijing, 100021 China

**Keywords:** CAR T cell therapy, Solid tumor, Tumor microenvironment, Antigen, Heterogeneity, T cell exhaustion, Toxicity, Combined therapies, Clinical trial

## Abstract

The application of chimeric antigen receptor (CAR) T cells in the management of hematological malignancies has emerged as a noteworthy therapeutic breakthrough. Nevertheless, the utilization and effectiveness of CAR-T cell therapy in solid tumors are still limited primarily because of the absence of tumor-specific target antigen, the existence of immunosuppressive tumor microenvironment, restricted T cell invasion and proliferation, and the occurrence of severe toxicity. This review explored the history of CAR-T and its latest advancements in the management of solid tumors. According to recent studies, optimizing the design of CAR-T cells, implementing logic-gated CAR-T cells and refining the delivery methods of therapeutic agents can all enhance the efficacy of CAR-T cell therapy. Furthermore, combination therapy shows promise as a way to improve the effectiveness of CAR-T cell therapy. At present, numerous clinical trials involving CAR-T cells for solid tumors are actively in progress. In conclusion, CAR-T cell therapy has both potential and challenges when it comes to treating solid tumors. As CAR-T cell therapy continues to evolve, further innovations will be devised to surmount the challenges associated with this treatment modality, ultimately leading to enhanced therapeutic response for patients suffered solid tumors.

## Background

Immunotherapies, specifically immune checkpoint inhibitors (ICIs) such as programmed cell death protein 1 (PD-1) and programmed cell death 1 ligand 1(PD-L1), provided a significant advance in cancer treatment [1]. CAR-T cell therapy is a novel form of immunotherapy that employs genetic engineering technology to modify T cells. This modification results in the expression of antigen receptor fragments on the cell surface, allowing for targeted antigen recognition and precise activation of T cells. Consequently, T cells can selectively attack specific targets by receiving antigen signals through the hinge and transmembrane regions [2]. CAR, as an engineered synthetic receptor, redirects T cells to identify the relevant antigens, thereby initiating anti-tumor effect that is not dependent on the major histocompatibility complex (MHC). Presently, CAR-T cell therapy has shown noteworthy and enduring effectiveness in the treatment of hematological malignancies [3]. Notably, two CAR-T cell products that target CD19, Kymriah and Yescarta, have received approval for the treatment of lymphoma or leukemia that is positive for CD19 [4].

The progress of CAR-T cell therapy in hematologic malignancies has spurred the development of CAR-T cell therapy for solid tumors, comprising more than 90% of all malignancies and posing a significant threat to global health. Currently, there are approximately 200 ongoing clinical trials investigating CAR-T therapy for solid tumors, yet the response rate has been unsatisfactory thus far [5], mainly due to the low specificity and high heterogeneity of tumor antigens, the immunosuppressive tumor microenvironment (TME), as well as limited T-cell infiltration and persistence [6]. This review outlined the evolution of CAR-T cell therapy and the major challenges it confronts in treating solid tumors. In addition, we discussed strategies and research directions to address these obstacles, while also offering an update on the progress of clinical trials of CAR-T cell therapy for solid tumors. The purpose of this article is to provide physicians and researchers working in the field with a useful resource.

## Development of CARs

**Fig. 1:**
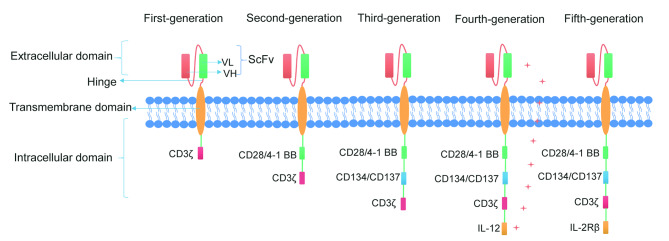
Five generations of CAR molecules

The CAR structure is primarily made up of three functional domains: the extracellular domain, the transmembrane domain, and the intracellular signal transduction domain [7]. The extracellular domain comprises the antigen recognition domain (single chain fragment variable, scFv) and the hinge portion, with scFv serving as the foundation for CAR’s specificity for tumor antigens, constructed from the peptide linkage of the variable light chain (VL) and the variable heavy chain (VH) of the antibody. The antigen recognition domain can directly identify tumor cell surface antigens, which in turn leads to MHC-independent T cell activation [8]. Another extracellular structural section known as the hinge region connects the scFv to the transmembrane structure and allows the antigen-binding structural section to overcome the spatial site barrier to reach the targeting epitope [9]. The intracellular domain has a direct impact on the CAR-activated T-cell effect, making it the most critical component of the CAR-T structure. The fusion between extracellular antigen-targeting structural domains and intracellular structural domains occurs via transmembrane structural domains. The first-generation CAR-Ts exclusively comprise CD3ζ without any co-stimulatory structural domains in the intracellular signaling domains. Despite their specific anti-tumor properties, first-generation CAR-Ts tend to be more cytotoxic, less persistent, and less effective [10]. The second-generation CAR, an evolution of the first-generation, incorporates an immunoreceptor tyrosine-based activation motif (ITAM) originated from co-stimulatory molecules like CD28 or CD137 (4-1BB) within the intracellular domain. This enhancement allows T cells to simultaneously receive both antibody stimulation signals and co-stimulatory signals when extracellular antigen recognition domains bind to target antigens [11, 12]. With this improvement, the second-generation CAR has demonstrated a significantly enhanced activation capability, resulting in improved therapeutic outcomes in the clinical treatment. The third-generation CAR is designed with an intracellular structural domain containing two co-stimulatory structural molecules, either CD28 and CD134 or CD28 and CD137 [13, 14]. Whereas fourth-generation and fifth-generation CAR-Ts have been developed upon the foundation of the 2G to further enhance anti-tumor responses and safety, with fourth-generation containing inducers for intracellular production of the target cytokines (IL-7, IL-12, IL-15, etc.) [15], while the fifth-generation having intracellular fragments of cytokine receptors like IL-2Rβ [16]. Presently available CAR-T cell products approved by the U.S. Food and Drug Administration (FDA) are based on second-generation designs. This is due to the fact that second-generation CAR-Ts are supported by a larger amount of clinical data, greater stability and more mature technology processes [17–19] (Fig. 1).

The CAR structure primarily comprises three functional domains: the extracellular domain, the transmembrane domain, and the intracellular domain. The first-generation CAR has an intracellular domain containing only CD3ζ, lacking co-stimulatory signals. The second-generation CAR includes a co-stimulatory domain like CD28 or 4-1BB, while the third-generation CAR consists of two or more co-stimulatory domains. The fourth-generation CAR, based on the second-generation CAR, incorporates the expression of specific cytokines, while the fifth-generation CAR adds co-stimulatory domains activating other signaling pathways.

## Challenges and coping strategies of tumor target antigens

Selecting suitable target antigens is the key to the efficacy of CAR-T cell therapy. Ideally, these antigens should possess attributes like high coverage, stability, and specificity. Nevertheless, there are currently two major concerns regarding target antigens for CAR-T cell therapy in solid tumors: the absence of appropriate tumor-specific antigens (TSAs) and the presence of antigen heterogeneity.

### The lack of appropriate TSA

Based on their expression patterns, tumor antigens are categorized as TSAs and tumor-associated antigens (TAAs). TAAs are present not only in tumors but also in normal tissues; in contrast, TSAs are solely expressed on tumor cells [20]. Due to the lack of TSAs, most of the current targets of CAR-T cell therapy for solid tumors are TAAs like the epidermal growth factor receptor (EGFR), human epidermal growth factor receptor 2 (HER2), mesothelin (MSLN), human endogenous retrovirus-H long terminal repeat-associating protein 2 (HHLA2), disialoganglioside GD2. Off-target effects are unavoidable due to TAAs’ limited specificity, which can occasionally have serious negative effects or even be fatal [21]. Hence, it is imperative to devise novel approaches for mitigating off-target toxicity caused by CAR-T cells.

### Heterogeneity of tumor target antigen

There is a high level of heterogeneity in tumors. It is noteworthy that significant differences in tumor antigen expression can occur within a patient’s identical anatomical region or even among the cells of the same tumor [22]. In addition, the quantity or type of tumor antigens varies between pre- and post-tumor therapy, as well as between primary and recurrent lesions [23]. Traditional CAR-T cells are limited to recognizing a single tumor surface marker, which leads to the escape of tumor cells that either lack or poorly express that particular antigen, ultimately resulting in tumor recurrence. Thus, the heterogeneity of tumor antigens raises the risk of tumor recurrence and makes solid tumor treatment more challenging.

To address the issues of off-target toxicity and tumor escape, which arise from the lack of suitable TSAs and heterogeneity of tumor antigens, several key strategies have been developed:

#### Pooled CAR-T cell therapy

Pooled CAR-T cell therapy entails the concurrent or sequential use of multiple distinct CAR-T cell types, each designed to target a specific antigen. By implementing this approach, the capacity of CAR-T cells to identify and attach to the corresponding tumor cells can be enhanced, thereby reducing the likelihood of tumor resistance. For instance, the combination of CAR-T cells respectively targeting EGFR and CD133 has been shown to improve the efficacy in cholangiocarcinoma [24]. In addition, research has shown that the co-application of prostate stem cell antigen (PSCA)-targeted and mucin 1 (MUC1)-targeted CAR-T cells effectively eradicated cancer cells in individuals diagnosed with PSCA + and MUC1 + non-small cell lung cancer (NSCLC) [25].

#### Bispecific CAR-T cell(dual CAR-T cell)

Bispecific CAR-T cells, also known as dual CAR-T cells, express two distinct CARs simultaneously on the surface of a single cell and demonstrate enhanced antitumor efficacy in comparison to Pooled CAR-T cells. For example, in a glioblastoma model, bispecific CAR-T cells co-expressing IL13Rα2 and HER2 CAR molecules demonstrated stronger anti-tumor ability and reduced tumor escape than monospecific or HER2 and IL13Rα2 pooled CAR-T cells [26]. Similarly, in an in vitro breast cancer model, ErbB2 and MUC1 biCAR-T cells demonstrated potent anti-tumor activity [27].

Dual CAR T-cells employ an AND-logic-gated strategy to effectively target dual-TAA-positive cancer cells, while showing no response to single-TAA-positive tumor cells. The effectiveness and safety of dCAR-T cells against pancreatic cancer cells expressing carcinoembryonic antigen (CEA) and MSLN have been confirmed [28].

#### TanCAR

The tandem CAR (TanCAR) combines two TAA-specific scFv along with an intracellular signaling component, forming a unified CAR on the cell surface. This design enables the simultaneous targeting of both antigens, thereby increasing anti-tumor activity. In an orthotopic glioblastoma (GBM) model, TanCAR targeting HER2 and IL13Rα2 exhibited greater efficacy compared to bispecific CAR-T cells [29].

### synNotch AND Gate T cell

The regulation of Notch signaling is involved in various aspects of tumor biology, including tumor angiogenesis, the maintenance of tumor stem cells, and the response of immune cells such as dendritic cells (DCs), T cells, and macrophages [30, 31]. The main triggering mechanism of the Notch signaling pathway involves several steps: (i) Following the transportation of the Notch receptor protein to the Golgi apparatus, it undergoes cleavage into two fragments and is subsequently transported to the cell surface to form heterodimers; (ii) Ligand from signaling cell binds to the Notch receptor extracellular domain (NECD), or activates Notch receptor without direct inter-cell contact to separate extracellular subunits from the transmembrane subunits of the receptor, thereby releasing the activated Notch receptor intracellular domain (NICD); (iii) The activated NICD enters the nucleus and forms transcription complexes with other proteins, thereby regulating gene transcription; (iv) In addition, the activated NICD can directly activate gene expression through irregular regulation [32, 33]. The SynNotch receptor contains the core regulatory domain from Notch but features a synthetic extracellular recognition domain and a synthetic intracellular transcription domain. Upon binding to the homologous antigen, the synNotch receptor undergoes induced transmembrane cleavage, similar to natural Notch activation, thereby releasing intracellular transcription domains into the nucleus and activating the expression of target genes regulated by the homologous upstream cis-activated promoter [34, 35]. Specifically, after stimulation at the initial target, SynNotch induces the expression of CAR in T cells. Then T cell activation ensues as the CAR binds to the antigen on target cells. T cell activation occurs only when tumor cells express two specific surface antigens simultaneously. Consequently, cells expressing only one antigen remain unaffected, effectively minimizing off-target toxicity. For instance, receptor tyrosine kinase-like orphan receptor 1 (ROR1)-CAR-T cells were modified to incorporate synNotch receptors with specificity for EpCAM or B7-H3, both present on ROR1 + tumor cells but not on ROR1 + stromal cells. This strategy resulted in tumor regression in ROR1 + tumor cells with reduced toxicity [36].

#### Universal CAR

The fifth-generation CARs, also referred to as the universal CARs, have been engineered to address the constraint where traditional CAR T cells target only one or two antigens. The innovation aims to increase flexibility and broaden antigen recognition. These advanced CARs use a “lock-key” system to divide the antigen-targeting domain and the T cell signaling unit, endowing CAR-T cells with nearly full antigen specificity [37].

#### On-Switch CAR

Switch on or off CAR-T cell has emerged as a new method to regulate the activity and mitigate potential toxicity. On-switch CAR separates the signal domain from the co-regulatory domain, and promotes the assembly of the two CAR fragments through the introduction of heterodimerized small molecules, which can activate T cells. This design enables the precise regulation of T cell activity, activation dose, time and location, thereby reducing first-pass toxicity and off-tumor toxicity [38, 39]. For instance, the novel CAR-T cell with a small bifunctional molecule “switch” composed of folic acid and fluorescein isothiocyanate exhibited heightened cytotoxicity specifically against folate receptor (FR)-positive cells while remaining inactive against FR-negative cells, thus enhancing the safety for CAR-T cell therapy [40]. Subsequent studies confirmed the effective, dose-dependent antitumor activity of On-switch CAR T in vivo using xenograft models, and demonstrated the advantages of dose-titration of On-switch CAR-T, which was beneficial to extending the application of CAR-T therapy to solid tumors [41]. Another preclinical study confirmed the anti-tumor efficacy of switchable CAR-T cells targeting HER2 in a pancreatic ductal adenocarcinoma xenograft model. The ability to modulate CAR-T activity through switchable dosing also presented a potential avenue for enhancing safety [42].

#### Suicide genes

Furthermore, an additional strategy to mitigate the off-target toxic response is incorporating suicide genes such as inducible caspase 9 (iCasp9) suicide gene and thus inducing the apoptosis of CAR-T cells that lead to a toxic response [43].

## Challenges and coping strategies of immunosuppressive microenvironment

### Tumor immunosuppressive microenvironment

The intricate immune inhibitory network within the TME, known as the tumor immunosuppressive microenvironment, is comprised of various immune cells, secretions and inhibitory signals, all working together to facilitate tumor initiation and progression [44]. The suppressive immune cells within the TME, including tumor-associated macrophages (TAMs), regulatory T cells (Tregs), and myeloid-derived suppressor cells (MDSCs), exert their inhibitory effects on proliferation and effective antitumor response of effector cells [45]. These immune cells and tumor cells promote the production of immune-suppressive-related cytokines, like TGF-β, IL-10, and IL-4, which accelerate the exhaustion of T cells and CAR-T cells [46]. CAR-T cells may exhibit increased PD-1 expression due to the TME. Consequently, the binding of PD-1 on CAR-T cells to PD-L1 on tumor cells initiates inhibitory signals, leading to compromised functionality of CAR-T cells [47] and facilitating immune evasion by tumor cells [48].

TAMs, as one of the major tumor-infiltrating immune cell types, can be classified into two distinct subtypes: classically activated M1 macrophages and alternatively activated M2 macrophages. These two functionally different types can interconvert when TME is altered. M1 macrophages are generally regarded as tumoricidal macrophages, primarily involved in anti-tumor activities and immune enhancement. In contrast, M2 macrophages can promote the secretion of IL-8 by Tregs, leading to the production of transforming growth factor beta (TGFβ), which subsequently inhibits anti-tumor immune responses [49]. Additionally, studies have shown that TAMs promote tumor growth through various mechanisms, including promoting angiogenesis, establishing a barrier to prevent the entry of effector T cells into the central tumor region, impeding the cross-presentation of tumor antigens by conventional dendritic cells, as well as facilitating tumor invasion and metastasis [50]. Hence, it is of great significance to develop practical approaches for depleting or reprogramming TAMs. Alfonso et al. developed a second-generation CAR that targeted the mouse pan-macrophage marker F4/80 (F4.CAR), meanwhile demonstrated that F4.CAR-T cells could selectively kill F4/80 + macrophages and eosinophils while locally release pro-inflammatory cytokines such as interferon-γ (IFNγ) and tumor necrosis factor (TNF) to enhance the anti-tumor effect within the tumor. This strategy has been shown to inhibit tumor progression and extend survival in a lung cancer model, as well as provide anti-tumor benefits in ovarian and pancreatic cancer models [51]. Additionally, reducing M2 macrophages and promoting the anti-tumor M1 phenotype by repolarizing M2 phenotype is an effective strategy. Folate-targeted Toll-like receptor 7 agonist (FA-TLR7-1 A) has been observed to bind to endosomal TLR7 and initiate signaling pathways to reprogram TAM/MDSCs into pro-inflammatory M1-like myeloid cells [52]. Based on this, Luo et al. further showed that the combined use of FA-TLR7-1 A and CAR-T cell therapy not only induced the repolarization of TAMs and MDSCs from an M2-like anti-inflammatory phenotype to an M1-like pro-inflammatory phenotype but also promoted CAR-T cells and endogenous T cells accumulation and activation within solid tumors, thereby significantly increasing the therapeutic efficacy of CAR-T cells [53].

MDSCs have been implicated in the poor response of solid tumor patients to immunotherapy. MDSCs suppress the immune response of effector T cells through diverse mechanisms, which involve the induction of Tregs, generation of reactive oxygen species, release of anti-inflammatory cytokines such as IL-10 and TGFβ, and exhaustion of essential amino acids required for T cell proliferation by inducing arginase and indoleamine 2,3-dioxygenase [54]. Furthermore, by producing matrix metalloproteinase-9 (MMP-9), MDSCs remodel the extracellular matrix (ECM), which promotes angiogenesis, tumor aggression, and spread [55]. Given these facts, researchers have been devoted to finding strategies to eliminate MDSCs from the TME. TNF-related apoptosis-inducing ligand-receptor 2 (TR2) is a receptor expressed on MDSCs that activates apoptosis upon binding to soluble ligands. Nalawade et al. developed TR2.41BB, a new chimeric co-stimulatory receptor, which encoded the scFv of the TR2 agonist antibody DS- 8273a followed by a 41BB endo-domain, and they validated it in three different breast cancer models to show that its co-expression on CAR-T cells augmented the anti-tumor effectiveness of CAR-T cells targeting MUC1 or HER2 [56].

TGFβ is a crucial mediator of immune suppression in most solid tumors. It restrains T cell activity by attaching to the TGFβRI and TGFβRII receptors, leading to decreased T cell proliferation, diminished cytokine generation, and impaired cytotoxic capability [57]. Additionally, TGFβ encourages T cells to differentiate into Tregs. Currently, several CAR approaches are under investigation to counteract TGFβ-mediated immune suppression. Carole et al. developed a TGFβ-switch receptor (TGFβ SwR) by combining the transmembrane and intracellular domains of IL-2/IL-15 receptor β and γ chains with the extracellular domains of TGFβ receptor I and II, aiming to transmit activating signals to T cells when triggered by TGFβ. They co-expressed the TGFβ SwR with CARs, demonstrating that this approach allowed CAR-T cells to maintain effective and persistent function in TGFβ-rich environments [58]. Preclinical studies have showed that inhibiting TGFβ signaling can be achieved by overexpressing dominant-negative TGFβRII20 [59] (TGFβRDN) or knocking out TGFBR2 using CRISPR-Cas9 [60]. Based on this preclinical evidence, Vivek et al. announced the findings of a Phase 1 trial on prostate-specific membrane antigen (PSMA)-targeting CAR T cells modified with a TGFβRDN. The clinical trial results revealed that TGFβRDN acted as a functional “armor”, effectively reducing common immune inhibitory barriers in the TME, increasing cytokine production, and improving CAR T cell persistence [61].

Recent studies have indicated that constructing TRUCKs or armored CARs, which secrete cytokines or express ligands in a constitutive or inducible manner, is an appealing option for enhancing the effectiveness of CAR-T cells by engaging in endogenous immune responses or creating an immune-supportive environment [16]. IL-12, an inflammatory cytokine, is believed to increase the cytotoxic capacity of CD8 + cells, facilitate antigen cross-presentation, reprogram MDSCs [62], and also alleviate tumor escape due to antigen loss by recruiting and activating macrophages [63]. In an ovarian cancer model, researchers have showed the effectiveness of IL-12 armored CAR-T cells in modifying the TME and overcoming the PD-L1-mediated suppression. This approach exhibited superior anti-tumor effects with favorable safety profiles [64]. Similar immune responses of IL-12 armored CAR-T cells have also been observed in hepatocellular carcinoma (HCC) [65]. IL-15 plays a crucial role in the survival of memory T cells and induces proliferation and activation of NK cells. Xu et al. confirmed in a mouse model that co-expression of IL-15 with CAR enhanced the in vivo persistence and efficacy of CAR-NKT without significant toxicity [66]. Furthermore, CAR-T cells modified with IL-18 have also been created. IL-18 cytokine creates a pro-inflammatory environment, recruiting bystander effector cells to the tumor location and enhancing their cytolytic activity [17]. Preclinical studies have verified that IL-18 TRUCKs CAR-T cells targeting GD2 induced monocytes recruitment, which helped reprogram the tumor stroma into a more favorable environment, thus making CAR-T cell therapy for solid tumors more effective [67]. These findings lay the foundation for further validation through subsequent clinical research.

The PD-1/PD-L1 axis is essential for the formation of inhibitory environments. PD-1 is expressed on various immune cell types, including natural killer (NK) cells, dendritic cells, CD4 + and CD8 + T lymphocytes and B lymphocytes. While the main cells that express PD-L1 are tumor cells, Treg cells, MDSCs, and TAMs. Immune suppression can result from PD-1 binding to PD-L1 since it can prevent T cell activation and cytokine generation [68, 69]. Moreover, it has the ability to limit the activity of phosphatidylinositol-3-kinase (PI3K) molecules and block CD28 signaling, which reduces T cell proliferation, tumor-killing potential, and cytokine release. Numerous studies have indicated that blocking the PD-1/PD-L1 axis is an effective strategy for enhancing CAR-T cells activity. In clinical practice, commonly employed strategies include blockade antibodies and gene silencing techniques. The combination of CAR-T cell therapy and ICIs holds significant potential as a treatment approach for solid malignancies. Experimental results from mouse models suggested that the activity and efficacy of anti-MSLN CAR-T [70] and anti-hPSMA CAR-T [71] cells could be enhanced through their combination with anti-PD-1 antibodies. Consistent with previous studies, the in vitro and in vivo studies conducted by Li and colleagues corroborated that anti-HER2 CAR-T cells combined with anti-PD-1 antibodies could secrete more IL-2 and IFN-γ, effectively killing tumor cells [72]. Yukiko et al. discovered that the combination of PD-L1 inhibitors with CAR-T cell therapy transformed the phenotype of TAMs into an M1-like subset that has no inhibitory effect on CAR-T cells through IFN-γ signaling and led to a depletion of CD163 + M2 macrophages, thus bolstering the anti-tumor efficacy of CAR-T cells [73]. The modification of CAR-T cells through gene silencing techniques to achieve PD-1 knockout has been mainly studied in vitro, with restricted investigation into the in vivo expansion and anti-tumor characteristics of PD-1 knockout CAR-T cells [74]. Zhou and colleagues produced CAR-T cells with silenced PD-1 expression by using short-hairpin RNA (shRNA)-mediated gene silencing technology. They observed that efficient silencing of PD-1 significantly inhibited the immunosuppressive effects of the TME in subcutaneous prostate and leukemia xenograft mouse models, enhancing tumor-killing effects and extending the activation duration of CAR-T cells [75].

### Inefficient migration and infiltration of CAR-T cells

In solid tumors, CAR-T cell therapy has exhibited suboptimal outcomes because of physical barriers and the immune-suppressive TME [76]. Physical barriers such as aberrant vasculature, dense ECM, and interstitial fluid pressure hinder CAR-T cell infiltration and migration within solid tumors [77]. Additionally, the existence of immune-suppressive cells, regulatory cytokines, and inhibitory molecules within the TME further exerts constraints on the expansion and effector capabilities of CAR-T cells [78]. Improving the trafficking of CAR-T cells within solid tumors has emerged as a major research focus in the field.

Chemokines and their receptors are pivotal factors in T cell migration, and the localized expression of chemokines at the tumor site tightly links to T cell infiltration, tumor control, and patient prognosis [79]. Research has shown that tumors can impede the infiltration of effector T cells and promote immune evasion by disrupting the chemokine-receptor network [80]. Regarding this mechanism, some studies have developed CAR-T cells that overexpressed chemokine receptors. For instance, it has been showed that CAR-T cells overexpressing the IL-8 receptors CXCR1 or CXCR2 had better tumor infiltration and anti-tumor effects [81]. Other chemokine receptor targets under investigation included CX3CR1 [82], CXCR5 [83], etc. The research has indicated that CAR-T cells overexpressing chemokine receptors exhibited enhanced tumor infiltration capability and lower off-tumor, on-target toxicity. Another strategy focuses on modifying CAR-T cells to locally deliver chemokines in order to recruit more immune cells. One commonly studied co-expression molecule is CXCL9. Modified CAR-T cells not only recruit immune cells that express its receptor, including DC cells, monocytes, NK cells, and T cells [84], but also promote the polarization of effector Th cells [85] and inhibit angiogenesis [86], resulting in a stronger anti-tumor effect. These effects have been validated in experiments in vivo and in vitro [87].

Recent research has suggested that the ability of therapeutic T cells to home back to target tissues may not solely rely on signals from local chemokine factors. Instead, it may be governed by crucial intrinsic cellular programs acquired during the ex vivo production of T cells. This finding presents a novel perspective for effective T cell immunotherapy. In their study, Hong et al. demonstrated the involvement of the ST3GAL1-βII-spectrin axis, an intrinsic cellular program, in controlling the tissue-specific migration of activated therapeutic T cells following intravenous infusion. Restraining the function of the negative regulatory factor ST3 β-galactoside α-2,3-sialyltransferase 1 (ST3GAL1), or restoring the expression of the lymphocyte function-associated antigen-1 (LFA-1)-associated cytoskeletal molecule βII-spectrin, could enhance chemokine-dependent and tissue-specific migration of CAR-T cells. Simultaneously, it reduced their non-specific sequestration in non-inflammatory tissues [88].

Structural and functional abnormalities of tumor vasculature not only contribute to the formation of the TME but also facilitate tumor progression, metastasis, immune suppression, and treatment resistance [89]. Tumors exhibit irregular shapes and diameters, and the pericyte coverage of tumor blood vessels is unstable, leading to the formation of abnormal vascular networks and subsequent microenvironmental hypoxia and acidosis. The resulting imbalance in the microenvironment and disruption of the ECM significantly hinder the penetration of anti-tumor lymphocytes into the tumor tissue [90]. Therefore, developing approaches to disrupt the existing vascular network and prevent the formation of new blood vessels in solid tumors seems to be a feasible strategy to promote the infiltration of CAR-T cells into tumor locations. Vascular endothelial growth factor (VEGF) is a crucial mediator of tumor angiogenesis. Research suggested that blocking VEGF signaling could transiently repair and remodel tumor vasculature, reprogram the TME, promote tumor vascular normalization, and enhance anti-tumor immune response [91, 92]. Further evidence demonstrated that blocking VEGF signaling promoted the migration of adoptively transferred T cells (ATCs) into malignancies, consequently improving the effectiveness of ATC-based immunotherapy [93]. Research conducted by Lanitis et al. has revealed that upregulation of VEGF-A in cancer cells competed with VEGFR2-targeting CAR-T cells, thereby diminishing the effectiveness of CAR-T therapy [94]. Therefore, combining anti-VEGF/R2 antibodies with VEGFR2 CAR-T therapy may be a suitable strategy. In fact, in B16 melanoma, co-administration of anti-VEGF-A antibodies in vivo has been found to promote the durability of CAR-T cells and improve tumor control. Additionally, anti-VEGF treatment has also been observed to increase the delivery and effectiveness of EGFRvIII CAR-T cells in GBM models [95].

In solid tumors, cancer cell nests are commonly encased within a dense fibrous matrix composed of high levels of collagen, fibronectin, and hyaluronic acid (HA). In addition, there is a diverse population of cancer-associated stromal cells (CASCs) that consists of mesenchymal stem cells (MSCs), alpha smooth muscle actin-positive myofibroblasts (αSMA+), and cancer-associated fibroblasts (CAFs) [96].

The ECM of tumors is essential for tumor growth and spread [97]. Excessive generation of ECM components, including hyaluronan, can lead to increased interstitial fluid pressure and the formation of physical barriers, protecting tumor cells from attack by immune effector cells [98]. Therefore, it may be feasible to promote immune cell trafficking and inhibit tumor growth by depleting HA. HA can be hydrolyzed by hyaluronidases, and PH20 has been found to possess high hyaluronidase activity. Recombinant PH20 protein has been shown to enhance the penetration of chemotherapy drugs and the infiltration of immune cells [99]. Zhao et al. discovered that co-expression of secreted PH20 significantly enhanced the degradation capacity of anti-MSLN CAR-T cells towards HA, leading to improved invasion efficiency of CAR-T cells and enhanced anti-tumor activity [100]. Furthermore, heparan sulfate proteoglycans (HSPGs) are another significant constituent of the ECM and can be degraded by heparinase (HPSE). In light of this, Caruana et al. engineered CAR-T cells expressing HPSE and showed their enhanced ability to degrade the ECM, promoting the migration of tumor T cells and anti-tumor activity [78].

CAFs are critical constituents of the TME, impeding T cell infiltration and recruiting other immunosuppressive cells, thereby creating a highly tumorigenic and immunosuppressive niche [101]. Fibroblast activation protein (FAP), a type II serine protease, is predominantly expressed on CAFs in most malignant solid tumors, with minimal expression observed in normal tissues. Therefore, FAP emerges as an attractive immunotherapeutic target [102]. Research has demonstrated that CAR-T cells targeting stromal cells expressing FAP effectively inhibited tumor growth in mouse models of solid tumors. This was achieved by clearing FAP + stromal cells, depleting the fibrotic tumor-associated matrix, and reprogramming the immunosuppressive microenvironment [103, 104].

Finally, unlike hematological malignancies, which exist in the peripheral compartments and can be targeted through intravenous infusion of co-stimulatory CAR-T cells, solid tumors are confined within immunosuppressive niches, impeding effective infiltration of T cells due to aberrant vascular systems and poor stromal barriers. Additionally, intravenous administration of T cells does not routinely infiltrate non-inflammatory tumor tissues; instead, they remain sequestered within non-target organs [105]. Therefore, some researchers believe that local delivery of CAR-T cells presents a potential solution to these limitations in cellular immunotherapy for solid tumors [106]. Preclinical studies have demonstrated that local administration led to a robust local immune response by accelerating the proliferation of CAR-T cells within tumors and deepening their infiltration [105]. Furthermore, locally delivered CAR-T cells underwent antigen activation and proliferation while upregulating chemokine expression, thereby promoting systemic circulation and effective infiltration into distant tumors [107]. In a phase I clinical trial conducted by Adusumilli et al., it was discovered that the use of MSLN-targeting CAR-T cells through intrapleural administration had the potential for effective treatment and the establishment of durable systemic circulation in patients with pleural mesothelioma. Importantly, the treatment did not show significant toxicity towards normal tissues known to express MSLN [5].

## Challenges and coping strategies of limited persistence

CAR-T cell exhaustion represents a state of dysfunction marked by different epigenetic, metabolic, and phenotypic features [6]. T cell exhaustion manifests as reduced effector function, distinct epigenetic and transcriptional gene signatures, persistent expression of several inhibitory receptors, defective cytokine production, increased expression of chemokines, and impaired proliferative capacity [108, 109]. Within the TME, CAR-T cell exhaustion is caused by constant exposure to tumor antigens, soluble immune regulatory molecules, hypoxic circumstances, and regulatory immune cells like dendritic cells and macrophages [110]. Hence, preventing or delaying CAR-T cell exhaustion during oncotherapy is of great concern.

Several studies have reported that prolonged antigen exposure led to functional exhaustion of CAR-T cells and rapid reduction in their numbers [111]. Hence, gaining a thorough comprehension of the molecular processes of T cell exhaustion during antigen stimulation can provide valuable insights for new T cell engineering strategies. Prinzing et al. reported on the role of DNA methyltransferase 3 A (DNMT3A) in mediating T cell factor 7 (TCF7) and lymphoid enhancer-binding factor 1 (LEF1) site methylation, along with other transcriptional regulatory factors that stably restrict T cell developmental potential under sustained antigen exposure conditions. They certified that CAR-T cells lacking DNMT3A retained the ability to proliferate and generate anti-tumor responses during prolonged tumor exposure [112]. Similarly, Good et al. established an in vitro model of antigen-driven CAR-T cell exhaustion and found that the modulation of transcription factors ID3 and SOX4 might contribute to preventing or delaying CAR-T cell exhaustion following long-term antigen exposure. Their study revealed that ID3 knockout or SOX4 knockout CAR-T cells showed enhanced cytotoxicity in vitro and reduced expression of genes associated with T cell dysfunction [113, 114].

Optimizing the molecular structure design of CARs is a common strategy for enhancing CAR-T cell functionality and persistence. It is well-known that co-stimulatory receptors are essential for the activation and proliferation of T cells [115]. CARs containing CD28 or 4-1BB co-stimulatory domains have been widely applied, and both have shown significant clinical efficacy. Some studies suggested that CAR-T cells carrying CD28 intracellular domains exhibited stronger effector function [116], while 4-1BB intracellular domains promoted greater CAR-T cell persistence [117]. Based on this, recent research has discovered that third-generation CARs, combining inducible T cell co-stimulator (ICOS) and 4-1BB intracellular signaling domains, manifested more effective and persistent results in solid tumor models compared to 4-1BB-based CARs. Another intriguing study discovered that incorporating the co-stimulatory receptor OX40 into the CAR structure reduced apoptosis of CAR-T cells by upregulating genes encoding members of the Bcl2 family. The OX40 signal not only increased the cytotoxicity of CAR-T cells but also reduced exhaustion markers, thereby improving CAR-T cell persistence [118]. Beyond modifying CAR co-stimulatory signals, engineering appropriate cytokines is also a strategy to enhance CAR-T cell persistence. Research suggested that modifying CAR T cells to secrete IL-7 enhanced CAR-T cell proliferation and survival. It could also prevent exhaustion by downregulating PD-1 expression, thereby increasing their potential clinical applicability [119]. IL-15, a survival-promoting cytokine, plays a crucial role in maintaining long-lasting CD8 + memory T cells in a state of homeostasis, while also demonstrating the capacity to inhibit activation-induced cell death (AICD). In a study by Lenka et al., co-expressing CAR with a membrane-bound chimeric IL-15 was employed to maintain a long-term memory stem cell phenotype in CAR-T cells [120].

It is widely recognized that the PD-L1 pathway downregulates anti-tumor immune responses, inhibits the cytotoxicity of T cells in the TME, and mediates T cell exhaustion [121]. Numerous studies have demonstrated that blocking the PD-1/PD-L1 axis could reverse CAR-T cell exhaustion and increase T cell-mediated anti-tumor effects. Cheng et al. designed an autocrine scFv PD-L1 antibody and showed that CAR-T cells engineered with this autocrine PD-L1 scFv antibody exhibited enhanced anti-tumor activity. They found that the autocrine PD-L1 scFv antibody reduced the expression of exhaustion markers PD-1, TIM-3, and CTLA-4 in an in vivo tumor model, thus preventing CAR-T cell exhaustion [122]. Furthermore, in vitro experiments have revealed that combined PD-1 blockade could preserve cytokine secretion and prevent AICD in CAR-T cells, thereby enhancing CAR-T cell persistence [123].

A Toll-like receptor 7 (TLR7) agonist has shown the capacity to rejuvenate exhausted CAR-T cells in vitro [124]. However, due to the systemic immune activation and associated toxicity of the TLR7 agonist, it has not received FDA approval [125]. To avoid systemic immune stimulation, Napoleon et al. utilized the abilities of recently designed universal CARs to bind fluorescein and internalize a fluorescein-TLR7 agonist conjugate via CAR-mediated endocytosis. They demonstrated that anti-fluorescein CAR-mediated uptake of a fluorescein-TLR7-3 conjugate selectively reactivated exhausted CAR-T cells without inducing systemic immune activation and associated systemic toxicity [126].

Runt domain-related transcription factor 3 (Runx3) is considered a key regulator in promoting CD8 + T cell maturation [127], cytotoxic T lymphocyte (CTL) differentiation [128], and the formation of tissue-resident memory T cells (Trm) [129]. A study suggested that overexpression of Runx3 promoted T cell persistence in a B16 melanoma mouse model [129]. Wang et al. generated CAR-T cells overexpressing Runx3 (Run-CAR-T cells) and observed an elevated presence of Run-CAR-T cells in peripheral blood and their enhanced accumulation in tumor tissues, indicating that co-expression of Runx3 enhanced the in vivo persistence of CAR-T cells [130].

## Challenges and coping strategies of toxicities

The lack of TSAs in CAR-T cell therapy raises the possibility of off-target impact. Furthermore, after infusion, the CAR-T cells that identify the target antigen can trigger an activation-induced release of cytokines or stimulate immune cells to release inflammatory cytokines, which may result in cytokine release syndrome (CRS), neurotoxicity, and various additional adverse effects. Section 3 has provided a thorough discussion on off-target toxicity, and this chapter primarily focuses on other toxicities.

### Cytokine release syndrome, CRS

CRS, an inflammatory response throughout the body caused by the excessive activation of effector cells and the release of numerous cytokines [131], is the most prominent and commonly observed toxicity associated with CAR-T cell therapy. It presents with various symptoms, such as fever and hypotension, etc. Studies of CRS have predominantly focused on hematologic malignancies. In these settings, CAR-T cells or other immune cells can generate the cytokines associated with CRS. the cytokines include IL-1, IL-6, IFN-γ, and granulocyte-macrophage colony-stimulating factor (GM-CSF), which are responsible for stimulating the growth of granulocyte-macrophage colonies [132]. CRS generally manifests during the first week after CAR-T cell therapy and has a relatively high incidence. It often presents as a few days of fever, and in some cases, symptoms can progress to include hypotension, hypoxia, organ dysfunction, and even life-threatening [132].

Extensive research has been conducted to investigate the mechanisms underlying CRS [133]. CAR-T cells, upon identifying tumor antigens, secrete a substantial quantity of perforin/granzyme and cytokines, resulting in tumor pyroptosis. The resultant pyroptotic products and pro-inflammatory factors recruit and activate macrophages, which serve as a crucial source of CRS-related inflammatory cytokines. Among the cytokines released by the monocyte-macrophage system, IL-6 has a crucial function in the initiation and advancement of CRS through both trans and cis signaling pathways mediated by JAK/STAT3. Additionally, IL-6 acts on endothelial cells via the cis signaling, stimulating the further release of pro-inflammatory molecules and thereby amplifying the inflammatory response in a cyclical manner. The severity of CRS is directly related to the increased expression of gasdermin (GSDM), which primarily determines the incidence of cell apoptosis or pyroptosis.

In certain clinical trials, Tocilizumab, a blocker of the IL-6 receptor, has demonstrated efficacy in decreasing or eradicating toxicities associated with CRS, and is commonly prescribed as first-line therapy in CRS [134, 135]. Tocilizumab is typically reserved for severe cases of CRS, while milder cases of CRS may be managed with supportive measures including hemodynamic monitoring and mechanical ventilation. According to recent research, the early use of tocilizumab and/or corticosteroids may decrease the occurrence of severe CRS after the administration of CD19 CAR-T cells, while still maintaining the effectiveness against tumors [136]. Corticosteroids are typically used to treat tocilizumab-resistant or severe (Grade 3 or 4) CRS [137]. Furthermore, IL-1 also plays a significant role in both CRS and neurotoxicity. Anakinra, a synthetic IL-1 receptor inhibitor, has been shown to effectively treat CRS in mouse models without impacting the growth of CAR-T cells [138]. Based on the findings in animal models, corresponding clinical trials are currently underway.

To avoid long-term adverse outcomes caused by low-dose infusion and intense toxic reactions associated with high-dose infusion, a stepwise dosing regimen is recommended for the administration of CAR-T cells. The approach includes a sequence of CAR-T cell administrations with escalating doses, and halts the infusion if early clinical signs of CRS are detected, which shows promise in achieving a balance between the efficacy and safety of CAR-T cell treatment [139].

Furthermore, enhancing the CAR framework has been shown to mitigate toxic reactions. Research has shown that CAR-T cells containing the 4-1BB co-stimulatory region demonstrated lower levels of toxicity in comparison to CAR-T cells possessing the CD28 co-stimulatory domain [140]. Knocking out critical cytokine genes such as GM-CSF [141] and IL-6 [142] has been demonstrated as viable solution to toxicity.

**Fig. 2:**
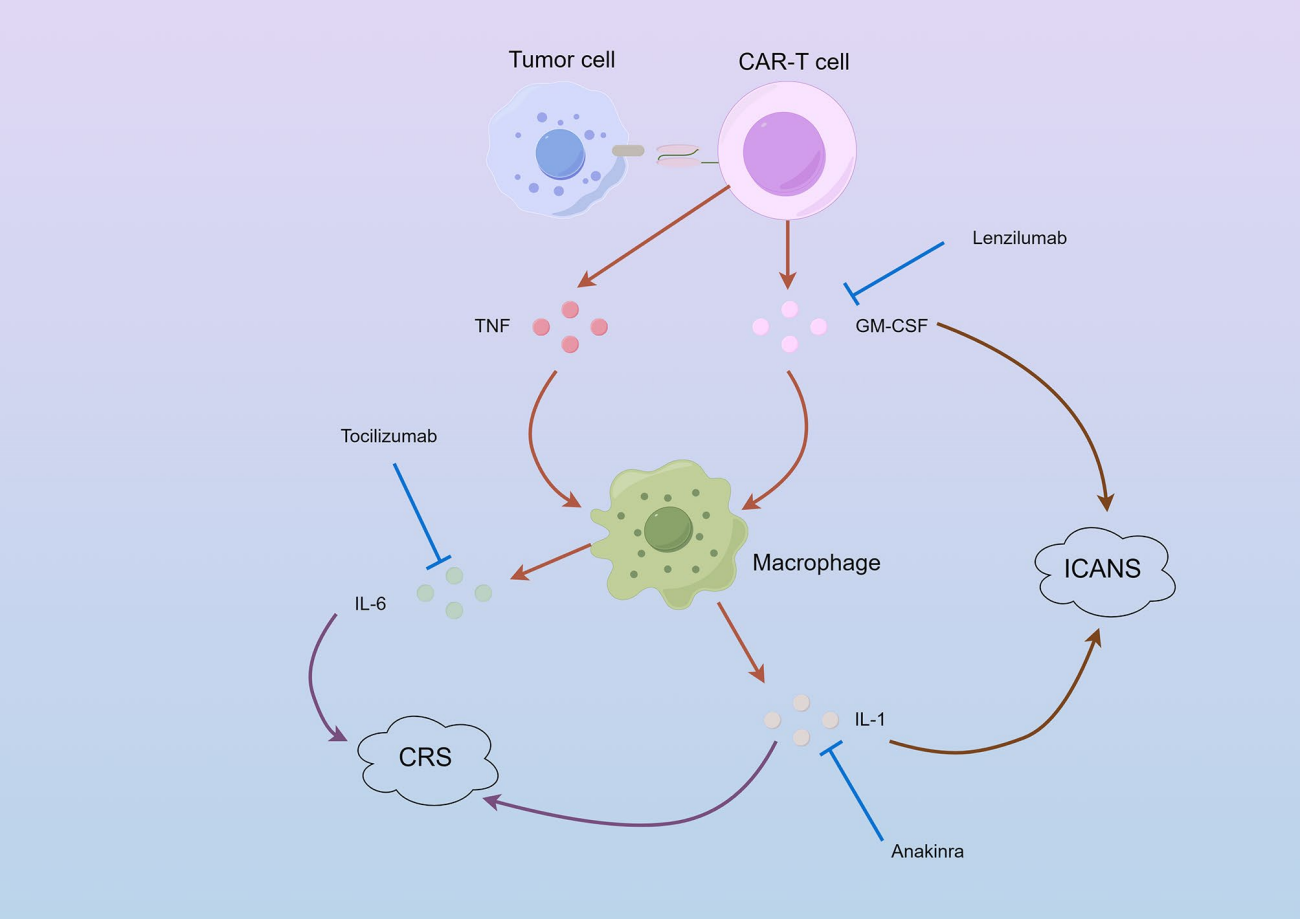
Mechanisms and potential therapeutic drugs of CRS and ICANS.

After recognition of tumor antigens by CAR-T cells, activation-induced cytokine release is triggered, including TNF, GM-CSF, etc. These pro-inflammatory factors recruit and activate macrophages, serving as crucial sources of inflammatory cytokines related to both CRS and ICANS. Cytokines released by the monocyte-macrophage system, such as IL-6 and IL-1, play pivotal roles in the initiation and progression of CRS. While IL-1 and GM-CSF are closely associated with ICANS. Therefore, inhibitors of the IL-6 receptor (such as Tocilizumab) and the IL-1 receptors (such as Anakinra) can alleviate CRS-related toxicity. Additionally, IL-1 receptor inhibitors (such as Anakinra) and anti-GM-CSF monoclonal antibodies (such as Lenzilumab) may play essential roles in combating ICANS toxicity.

### Immune effector cell-associated neurotoxicity syndrome, ICANS

Neurotoxicity, which manifests as a toxic encephalopathy with various neurological and psychiatric symptoms, is the second most common form of toxicity associated with CAR-T cell therapy. The American Society for Transplantation and Cellular Therapy (ASTCT) has defined “immune effector cell-associated neurotoxicity syndrome” (ICANS) [132]. The clinical manifestations of ICANS are diverse, including somnolence, disorientation, and aphasia [143]. In more severe cases, cognitive impairment, seizures, and fatal cerebral edema can also occur [137]. Most ICANS cases occur concomitantly with or subsequent to CRS, but there are instances where ICANS can arise independently without CRS toxicity, suggesting that ICANS may have distinct pathophysiology mechanisms from CRS and requires different management approaches [134] (Fig. 2).

At present, there is a restricted comprehension regarding the mechanisms of ICANS. The blood-brain barrier (BBB), a complex structure consisting of tightly linked endothelial cells, endothelial basement membrane, pericytes encircling the capillaries, parenchymal basement membrane, and astrocytic foot processes, serves as a barrier to the entry of molecules and cells into the central nervous system (CNS) [144]. During an inflammatory condition, the activation of cerebral endothelial cells causes disruption to the integrity of BBB and results in heightened vascular permeability. This leads to the buildup of CNS cytokines, as well as host immune cells like T lymphocytes and activated monocytes, in addition to CAR-T lymphocytes. This cumulative effect serves as a trigger for inflammation within the CNS. This leads to neuronal damage, functional impairments, and the onset of a range of psychiatric symptoms [133]. The analysis of single-cell sequencing of cells associated with ICANS has revealed that GM-CSF is the factor most strongly linked to ICANS. Furthermore, recent studies have suggested that CD19 CAR-T cells specifically attacked brain barrier cells expressing CD19, resulting in the disruption of the BBB [145]. The high occurrence of ICANS linked to CD19 CAR-T cell therapy could be partly attributed to this.

Several studies have been conducted to explore strategies to address ICANS. In the first-line management of ICANS, corticosteroids exhibit powerful non-specific anti-inflammatory properties [137]. Dexamethasone is the most commonly used corticosteroid, due to its excellent CNS penetration capability [146]. Early intervention with corticosteroids has been found to reduce the occurrence of severe ICANS while maintaining CAR-T cell proliferation [147]. Anakinra, the recombinant IL-1 receptor antagonist, has shown the capability to cross the BBB and specifically act on IL-1β in the CNS, providing relief from ICANS symptoms [148]. GM-CSF is a crucial cytokine associated with ICANS, and Lenzilumab, an anti-GM-CSF monoclonal antibody, effectively mitigated CAR-T cell-induced ICANS in murine models by decreasing the infiltration of myeloid and T cells in the CNS [149].

## Combination therapies to enhance CAR‑T cells efficacy

Currently, numerous studies have suggested that the use of CAR-T cell therapy alone has shown limited efficacy in treating solid tumors. The combination of therapies presents possible approaches to enhance the efficacy of CAR-T cell therapy.

### Combination with chemotherapy

Research has indicated that the effectiveness of CAR-T cell therapy was improved by lymphodepletion [150]. Numerous approaches have been suggested to elucidate the curative advantages of lymphodepletion before the implementation of adoptive T-cell immunotherapy [151]. (1) Temporary ‘room’ for the infused cells is created by nonmyeloablative chemotherapeutic regimens. (2) By eliminating endogenous lymphocytes, infused T-cells can more effectively obtain crucial cytokines such as IL-2, IL-7, and IL-15, which promote their growth and viability. (3) Lymphodepletion has been reported to eradicate immunosuppressive cells such as regulatory T cells (Tregs) and myeloid-derived suppressor cells (MDSCs), while enhancing the effectiveness of cells that present antigens. (4) Certain lymphodepleting agents can counteract immune suppression by downregulating enzymes involved in the metabolism of tryptophan, particularly indoleamine 2, 3-dioxygenase (IDO). (5) Adoptively transferred T-cells exhibit improved tumor trafficking capability following lymphodepletion. In CAR-T cell therapy, lymphodepletion regimens like cyclophosphamide and fludarabine are frequently employed as chemotherapy agents [152]. The advantages of combination therapy have been substantiated in numerous studies. In a preliminary investigation of CD19 CAR-T cell treatment for recurring/insensitive chronic lymphocytic leukemia (CLL) and other B-cell tumors, it was discovered that cyclophosphamide-conditioning chemotherapy boosted the longevity of CAR-T cells and indicated potential enhancement in effectiveness [153]. Srivastava et al. demonstrated that the use of chemotherapy drugs to deplete lymphocytes could induce immunogenic cell death (ICD) and promote the release of chemokines that attracted T cells, leading to a significant increase in the infiltration of CAR-T cells into tumors [154].

TME is a significant barrier to CAR-T cell therapy efficacy. Chemotherapy has the ability to alter TME and enhance the effectiveness of CAR-T cell therapy. Docetaxel [155] and gemcitabine [156, 157], employed as neoadjuvant therapies, have shown the ability to enhance the effectiveness of GD2 CAR-T cells by decreasing MDSCs in the tumor. Doxorubicin has shown the capacity to boost the activity of CAR-T cells by decreasing the expression of PD-L1 on osteosarcoma cancer cells [158]. Studies have also demonstrated that oxaliplatin altered the TME by regulating the chemokine profile, resulting in increased recruitment of ROR1-CAR T cells [154]. Furthermore, in a phase I clinical trial [159], the exploration of a combined therapy involving CAR-T cells along with paclitaxel and cyclophosphamide revealed noteworthy clinical improvements in 21 of the 28 patients who had previously experienced unsuccessful paclitaxel therapy.

### Combination with radiotherapy

A key issue in the progression and immune evasion of solid tumors is the polarization of the TME towards an immune-suppressive and tolerant phenotype [160], leading to the transition of “hot” tumors to “cold” tumors. In addition to hindering the movement of CAR-T cells towards the center of the tumor, cold tumors also attract immune-suppressing cells, leading to the depletion of effector cells.

In the past few years, numerous studies have demonstrated that radiotherapy (RT) could transform “cold” tumors into “hot” tumors, allowing immune cells to penetrate tumor tissues and reshaping the immunosuppressive barriers in the local TME [161]. Hence, the fusion of RT and CAR-T treatment arises as a possibly advantageous approach to enhance the anti-cancer impact. Real-time imaging analysis in a glioblastoma model has shown that RT enabled the swift movement of CAR-T cells from the vascular system into the TME, as well as their amplification within the TME, leading to enhanced and prolonged immune responses [162]. Moreover, data from a preclinical study in mice models of glioma suggested that the combination of local tumor irradiation and NKG2D CAR-T cell therapy resulted in a synergistic impact. This was achieved by facilitating the movement of CAR-T cells towards tumor tissues and augmenting their effector capabilities [163].

### Combination with oncolytic viruses

Oncolytic viruses (OVs) are regarded as a type of tumor immunotherapy due to their potential impact on immune activation and virus-mediated tumor cell death [164]. OVs therapies, including oncolytic adenovirus (OAV), face several challenges, such as poor infiltration ability, antiviral immune response, off-target infection, inhibitory TME, and lack of specific targets [165]. Given these limitations, the benefits of OV monotherapy remain limited and insufficient for tumor clearance. Nevertheless, it can function as a supplementary approach to enhance the anti-cancer impacts of alternative therapies, such as CAR-T cell therapy. Conceptually, OVs can alleviate the obstacles of CAR-T cell therapy by many ways including: (1) direct tumor cells lysis and release of tumor neoantigens; (2) activation of local host innate immune responses, and subsequent cytokine release and recruitment of various immune cells; (3) carrying viral-encoded transgenes to ‘reprogram’ TME into a pro-inflammatory environment [166]. Mechanisms mentioned above have been supported by several recent in vitro and in vivo studies. A study utilized a combination of OAVs arming chemokine (C-C motif) ligand5 (CCL5) and IL-12 (Ad5-ZD55-CCL5-IL12) along with CAR-T cells that targeted carbonic anhydrase 9 (CA9) in renal cancer cells. The results demonstrated that Ad5-ZD55-CCL5-IL12 facilitated the infiltration, reproduction, and stimulation of CAR-T cells [167]. In addition, single-cell sequencing and spatial transcriptome analysis of animal tumor specimens have confirmed that OncoViron, a chimeric OAV, could not only induce substantial changes in the gene expression pattern of infected tumor cells but also recruit a considerable quantity of lymphocytes, NK cells, and mononuclear macrophages to the TME. Moreover, it induced the transformation of macrophages from M2 to M1 and stimulated the increased release of immune cell factors, thereby reshaping the TME and synergistically augmenting the effectiveness of CAR-T cell treatment [168].

In addition, Extensive studies have shown promising outcomes when CAR-T therapy is combined with ICIs, as elaborated in Sects. "Challenges and coping strategies of immunosuppressive microenvironment" and "Challenges and coping strategies of limited persistence".

## Clinical trials of CAR-T therapy for solid tumors

**Fig. 3:**
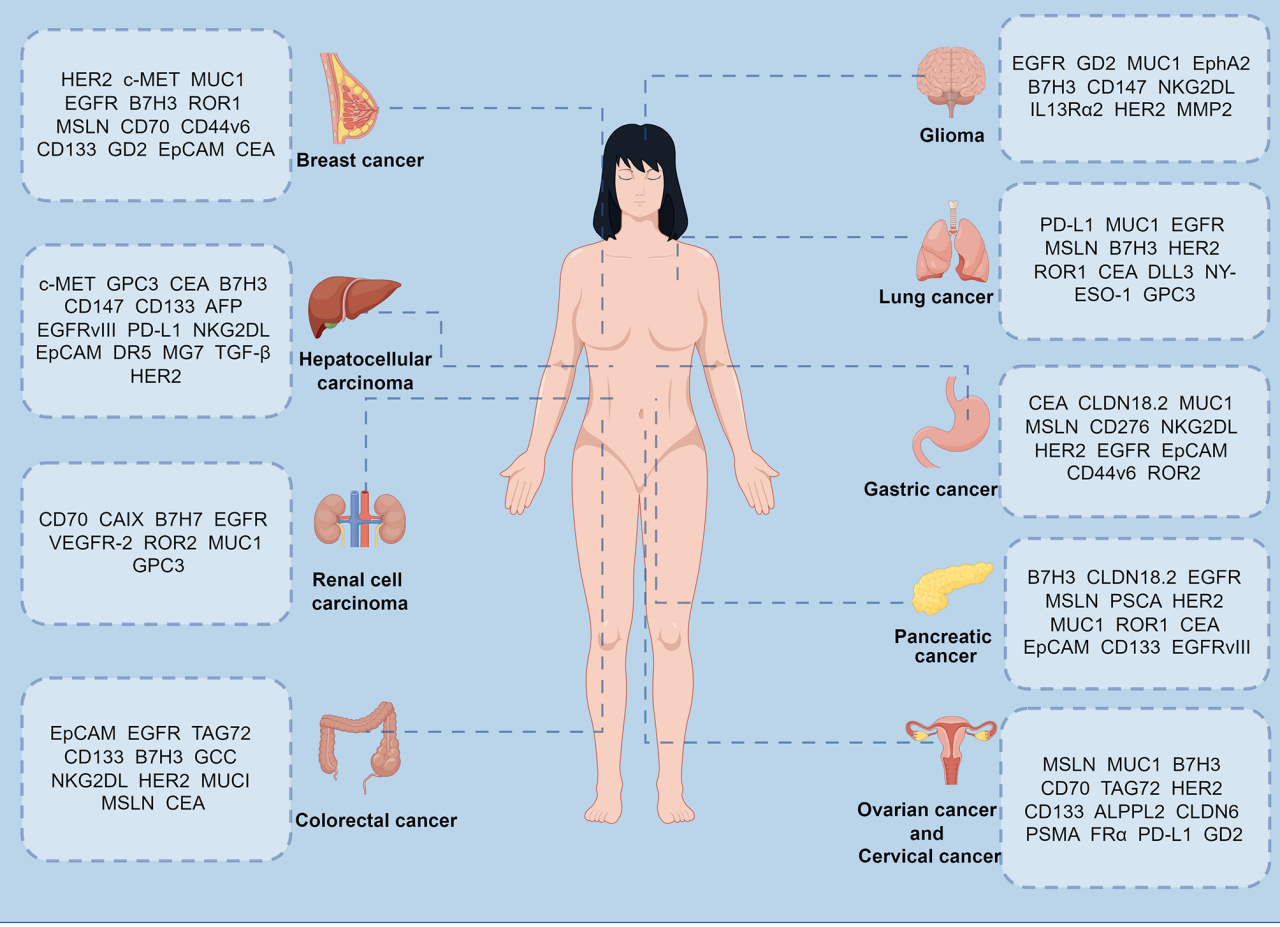
CAR-T therapeutic targets for common solid tumors

Extensive research has been conducted on CAR-T cell therapy in various types of solid tumors through clinical trials. This section provided an overview of the main targets of CAR-T cell therapy for specific solid tumors (Fig. 3), along with the corresponding clinical trial findings (Table 1). Our summary indicated that the majority of these clinical trials are in early stages, with most being initiated by research institutions such as hospitals and universities, reflecting a slower pace of commercialization of CAR-T cell therapy in solid tumors compared to hematologic malignancies.

**Table 1:** Major clinical trials of CAR-T cell therapy for solid tumors

Type of cancer	Target	NCT	n	Year	Phase	Company	Status
Lung cancer	EGFR	NCT05060796	11	2019	I	*NR (sponsor: Second Affiliated Hospital of Guangzhou Medical University)	Recruiting
		NCT05060796	11	2019	Early I	NR (sponsor: Second Affiliated Hospital of Guangzhou Medical University)	Recruiting
	HER2	NCT03740256	45	2020	I	NR (sponsor: Baylor College of Medicine)	Recruiting
		NCT01935843	10	2013	I/II	NR (sponsor: Chinese PLA General Hospital)	Unknown
	CEA	NCT02349724	75	2015	I	NR (sponsor: Southwest Hospital, China)	Unknown
		NCT04348643	40	2020	I/II	Chongqing Precision Biotech Co., Ltd	Recruiting
	MSLN	NCT03054298	27	2017	I	NR (sponsor: University of Pennsylvania)	Recruiting
		NCT01583686	15	2012	I/II	NR (sponsor: National Cancer Institute, NCI)	Terminated
	MUC1	NCT02587689	60	2015	I/II	PersonGen BioTherapeutics (Suzhou) Co., Ltd.	Unknown
		NCT03525782	20	2018	I/II	NR (sponsor: Hospital of Guangdong Pharmaceutical University)	Recruiting
	PD-L1	NCT02862028	20	2016	I/II	NR (sponsor: Shanghai International Medical Center)	Unknown
	ROR1	NCT02706392	60	2022	I	NR (sponsor: Fred Hutchinson Cancer Center)	Recruiting
	GPC3 or TGFβ	NCT03198546	30	2017	I	NR (sponsor: Hospital of Guangzhou Medical University)	Recruiting
	NY-ESO-1 or EGFR V III	NCT03638206	73	2018	I/II	Shenzhen BinDeBio Ltd.	Recruiting
	MAGE-A1, MAGE-A4, MucI, GD2, and MSLN	NCT03356808	20	2017	I/II	Shenzhen Geno-Immune Medical Institute	Recruiting
	HER2, MSLN, PSCA, MUC1, Lewis-Y, GPC3, AXL, EGFR, Claudin18.2/6, ROR1, GD1, or B7-H3	NCT04842812	40	2021	I	NR (sponsor: Hospital of Guangzhou Medical University)	Recruiting
Breast cancer	HER2	NCT04650451	220	2020	I	Bellicum Pharmaceuticals	Recruiting
		NCT03740256	45	2018	I	NR (sponsor: Baylor College of Medicine)	Recruiting
		NCT03696030	39	2018	I	NR (sponsor: City of Hope Medical Center)	Recruiting
	ROR1	NCT02706392	60	2016	I	NR (sponsor: Fred Hutchinson Cancer Center)	Recruiting
	c-MET	NCT01837602	6	2013	I	NR (sponsor: University of Pennsylvania)	Completed
		NCT03060356	77	2017	I	NR (sponsor: University of Pennsylvania)	Terminated
	MSLN	NCT02792114	36	2016	I	NR (sponsor: Memorial Sloan Kettering Cancer Center)	Recruiting
		NCT02414269	179	2015	I	NR (sponsor: Memorial Sloan Kettering Cancer Center)	Recruiting
	MUC1	NCT02587689	20	2015	I	PersonGen BioTherapeutics (Suzhou) Co., Ltd.	Unknown
	CD44v6	NCT04427449	100	2020	I/II	Shenzhen Geno-Immune Medical Institute	Recruiting
	EpCAM	NCT02915445	30	2016	I	NR (sponsor: Sichuan University)	Recruiting
	CD133	NCT02541370	20	2015	I	NR (sponsor: Chinese PLA General Hospital)	Completed
	CD70	NCT02830724	2	2016	I/II	NR (sponsor: National Cancer Institute, NCI)	Suspension
	CEA	NCT02349724	75	2015	I	NR (sponsor: Southwest Hospital, China)	Unknown
		NCT04348643	40	2020	I/II	Chongqing Precision Biotech Co., Ltd	Recruiting
	NKG2D	NCT04107142	10	2019	I	CytoMed Therapeutics Pte Ltd	Not yet recruiting
	GD2	NCT03635632	94	2018	I	NR (sponsor: Baylor College of Medicine)	Recruiting
Gastric cancer	EpCAM	NCT03563326	40	2018	I	NR (sponsor: Jian-Kun Hu, West China Hospital)	Unknown
	HER2	NCT04650451	220	2020	I	Bellicum Pharmaceuticals	Suspended
		NCT03740256	45	2018	I	NR (sponsor: Baylor College of Medicine)	Recruiting
	CEA	NCT04348643	40	2020	I	Chongqing Precision Biotech Co., Ltd	Recruiting
	Claudin18.2	NCT04581473	192	2020	I/II	CARsgen Therapeutics Co., Ltd.	Recruiting
		NCT04404595	110	2020	I/I	CARsgen Therapeutics Co., Ltd.	Recruiting
	MUC1	NCT05239143	100	2022	I	Poseida Therapeutics, Inc.	Recruiting
	MSLN	NCT03941626	50	2019	I/II	Shenzhen BinDeBio Ltd.	Unknown
	B7H3	NCT04864821	24	2021	I	PersonGen BioTherapeutics (Suzhou) Co., Ltd.	Unknown
	EGFR	NCT03740256	45	2018	I	NR (sponsor: Baylor College of Medicine)	Recruiting
	NKG2DL	NCT04550663	10	2020	I	NR (sponsor: The Nanjing Drum Tower Hospital)	Unknown
Liver Cancer	GPC3	NCT03198546	30	2017	I	NR (sponsor: Hospital of Guangzhou Medical University)	Recruiting
	CEA	NCT02416466	8	2015	I	NR (sponsor: Roger Williams Medical Center)	Completed
	CD133	NCT02541370	20	2015	I/II	NR (sponsor: Chinese PLA General Hospital)	Completed
	AFP	NCT03253289	13	2017	I	NR (sponsor: Tannaz Armaghnay)	Active, not recruiting
	EGFRvIII	NCT03941626	10	2019	I	Shenzhen BinDeBio Ltd.	Active, not recruiting
	B7H3	NCT05323201	15	2022	I/II	NR (sponsor: The Affiliated Hospital of Xuzhou Medical University)	Recruiting
	EpCAM	NCT03013712	60	2017	I/II	NR (sponsor: First Affiliated Hospital of Chengdu Medical College)	Unknown
	MUC1	NCT04842812	40	2021	I	NR (sponsor: Hospital of Guangzhou Medical University)	Recruiting
	NKG2DL	NCT04550663	10	2020	I	NR (sponsor: Nanjing Drum Tower Hospital of Nanjing University Medical School)	Unknown
	PD-L1	NCT03672305	50	2018	Early 1	NR (sponsor: The Second Hospital of Nanjing Medical University)	Unknown
	CD147	NCT03993743	30	2019	I	NR (sponsor: Xijing Hospital)	Unknown
Pancreatic cancer	MSLN	NCT02159716	19	2014	I	NR (sponsor: University of Pennsylvania)	Completed
		NCT03198546	30	2017	I	NR (sponsor: Hospital of Guangzhou Medical University)	Recruiting
	EGFR	NCT01869166	60	2013	I/II	NR (sponsor: Chinese PLA General Hospital)	Unknown
	HER2	NCT01935843	10	2013	I/II	NR (sponsor: Chinese PLA General Hospital)	Unknown
	CEA	NCT02850536	5	2016	I	NR (sponsor: Roger Williams Medical Center)	Completed
	CD133	NCT02541370	20	2015	I/II	NR (sponsor: Chinese PLA General Hospital)	Completed
	B7H3	NCT05143151	10	2021	I/II	NR (sponsor: Shenzhen University General Hospital)	Recruiting
	PSCA	NCT03267173	10	2017	I	NR (sponsor: Hospital of Harbin Medical University)	Unknown
	MUC1	NCT05239143	100	2022	I	Poseida Therapeutics, Inc.	Recruiting
	EpCAM	NCT05028933	48	2021	I	NR (sponsor: Zhejiang University)	Recruiting
	ROBO1	NCT03941457	9	2019	I/II	Asclepius Technology Company Group (Suzhou) Co., Ltd.	Unknown
Colorectal cancer	CEA	NCT02349724	75	2015	I	NR (sponsor: Southwest Hospital, China)	Unknown
		NCT02850536	5	2016	I	NR (sponsor: Roger Williams Medical Center)	Completed
		NCT04348643	40	2020	I/II	Chongqing Precision Biotech Co., Ltd	Recruiting
		NCT02959151	20	2016	I/II	Shanghai GeneChem Co., Ltd.	Unknown
	MSLN	NCT05089266	30	2021	I	Shanghai Cell Therapy Group Co.,Ltd	Not yet recruiting
		NCT04503980	10	2020	I	Shanghai Cell Therapy Group Co.,Ltd	Unknown
	EpCAM	NCT05028933	48	2021	I	NR (sponsor: Zhejiang University)	Recruiting
	MUC1	NCT05239143	100	2022	I	Poseida Therapeutics, Inc.	Recruiting
		NCT02617134	20	2015	I/II	PersonGen BioTherapeutics (Suzhou) Co., Ltd.	Unknown
	HER2	NCT03740256	45	2018	I	NR (sponsor: Baylor College of Medicine)	Recruiting
	B7-H3	NCT05190185	18	2022	I	PersonGen BioTherapeutics (Suzhou) Co., Ltd.	Recruiting
	EGFR	NCT03542799	20	2018	I	NR (sponsor: Shenzhen Second People’s Hospital)	Unknown
		NCT03152435	20	2017	I/II	NR (sponsor: Shenzhen Second People’s Hospital)	Unknown
	CD133	NCT02541370	20	2015	I/II	NR (sponsor: Chinese PLA General Hospital)	Completed
	c-Met	NCT03638206	73	2018	I/II	Shenzhen BinDeBio Ltd.	Unknown
Esophageal cancer	HER2	NCT03740256	45	2018	I	NR (sponsor: Baylor College of Medicine)	Recruiting
	EpCAM	NCT03013712	60	2017	I/II	NR (sponsor: Hospital of Chengdu Medical College)	Unknown
	NY-ESO-1	NCT03941626	50	2019	I/II	Shenzhen BinDeBio Ltd.	Unknown
	MUC1	NCT03706326	20	2018	I/II	NR (sponsor: Hospital of Guangdong Pharmaceutical University)	Unknown
Ovarian cancer	MSLN	NCT03615313	50	2018	I/II	Shanghai Cell Therapy Research Institute	Unknown
	FRα	NCT03585764	18	2018	I	NR (sponsor: University of Pennsylvania)	Recruiting
	HER2	NCT04660929	48	2020	I	Carisma Therapeutics, Inc.	Recruiting
	ALPPL2	NCT04627740	20	2020	I/II	NR (sponsor: Xinqiao Hospital of Chongqing)	Not yet recruiting
	B7H3	NCT04670068	21	2020	I	NR (sponsor: UNC Lineberger Comprehensive Cancer Center)	Recruiting
	TAG72	NCT05225363	33	2022	I	NR (sponsor: City of Hope Medical Center)	Recruiting
	MUC1	NCT05239143	100	2022	I	Poseida Therapeutics, Inc.	Recruiting
	CD70	NCT02830724	124	2016	I/II	NR (sponsor: National Cancer Institute, NCI)	Recruiting
Glioma	IL13Rα2	NCT02208362	82	2014	I	NR (sponsor: City of Hope Medical Center)	Active, not recruiting
		NCT00730613	3	2008	I	NR (sponsor: City of Hope Medical Center)	Completed
	EGFRvIII	NCT02209376	11	2014	I	NR (sponsor: University of Pennsylvania)	Terminated
	HER2	NCT03500991	48	2018	I	NR (sponsor: Seattle Children’s Hospital)	Recruiting
	EphA2	NCT03423992	100	2018	I	NR (sponsor: Xuanwu Hospital, Beijing)	Unknown
	MUC1	NCT02617134	20	2015	I/II	PersonGen BioTherapeutics (Suzhou) Co., Ltd.	Unknown
	CD147	NCT04045847	31	2019	Early I	NR (sponsor: Xijing Hospital)	Unknown
	MMP2	NCT04214392	36	2020	I	NR (sponsor: City of Hope Medical Center)	Recruiting
Head and neck squamous cell carcinoma	HER2	NCT03740256	45	2018	I	NR (sponsor: Baylor College of Medicine)	Recruiting
	MUC1	NCT05239143	100	2022	I	Poseida Therapeutics, Inc.	Recruiting

* NR: not reported

### Lung cancer

EGFR, MSLN, MUC1, CEA, PD-L1, ROR1, HHLA2 (also referred to as B7H7), HER2, and various other target antigens are being studied for CAR-T therapy in lung cancer. These antigens have undergone scrutiny in both preclinical and clinical trials, seeking to shed light on their suitability for CAR-T therapy in lung cancer.

EGFR is abundantly present on the surface of numerous solid cancer cells and is closely associated with the development and advancement of tumors [169]. Currently, there are several ongoing early-stage clinical trials (NCT01869166, NCT03182816, NCT04153799) examining the use of EGFR-targeted CAR T cell therapies in the treatment of lung cancer. In one of the Phase I clinical trials, individuals with relapsed/refractory NSCLC and positive for EGFR, were administered EGFR-CAR-T cell therapy and showed good tolerance to the treatment. Fever (grades 1 to 3) was the most frequent adverse event (AE), no grade 4 AEs or severe CRS were reported. Regarding efficacy, 1 patient achieved partial response (PR) that endured for over 13 months, 6 patients maintained stable disease (SD), and 2 patients encountered disease progression. Overall, the 9 patients experienced a median progression-free survival (mPFS) of 7.13 months and a median overall survival (mOS) of 15.63 months [170].

MSLN, a glycoprotein involved in cell adhesion, facilitates the invasion and metastasis of tumors [171]. Prior investigations have unveiled a positive correlation between MSLN overexpression in lung cancer patients and unfavorable prognoses [172]. In vivo studies have shown that MSLN-CAR-T cells displayed enhanced abilities to eliminate cancer cells in NSCLC when compared to conventional T cells [173]. On the basis of preclinical trials, Several clinical trials, including NCT02414269 and NCT02580747, are currently in progress for lung cancer using MSLN-CAR-T cells; however, comprehensive results data have yet to be disclosed.

MUC1, a transmembrane protein, plays a crucial role in cancer cell adhesion and metastasis [174]. Extensive studies have highlighted the markedly increased presence of MUC1 in lung cancer specimens compared to normal tissues [175]. Preclinical trials by Wei et al. combined CAR-T cells targeting PSCA with CAR-T cells targeting MUC1 in a patient-derived NSCLC xenograft mouse model, and the tumor growth was effectively inhibited [25]. Numerous clinical trials (NCT02587689, NCT03356808, NCT03198052, NCT03525782, NCT04842812) are currently underway, with the aim of utilizing CAR-T cells targeting MUC1 to treat lung cancer.

ROR1, a receptor similar to tyrosine kinase, exhibits increased expression in tumor tissues while showing minimal expression in normal tissues [176]. Notably, Wallstabe et al. have provided compelling evidence that ROR1-targeted CAR-T cell therapy effectively eliminated NSCLC tumor cells using a three-dimensional tumor model [177].

Furthermore, numerous researchers around the globe are actively engaged in clinical investigations pertaining to HER2-targeted CAR-T cells (NCT03198052, NCT01935843, NCT02713984, NCT03740256, and NCT04660929), PD-L1-targeted CAR-T cells (NCT03060343, NCT03330834), CEA-targeted CAR-T cells (NCT02349724 and NCT04348643), as well as B7-H3-targeted CAR-T cells (NCT04864821 and NCT03198052) in lung cancer. Moreover, several additional antigenic markers, such as CXCR4, PSCA, GPC3 (Glypican-3), EphA2 tyrosine kinase receptor, CD44v6, folate receptors (FRα and FRβ), Lewis-Y antigen, IL-13Rα2, L1 cell adhesion molecule, and GD2, are presently undergoing preclinical evaluation. However, it’s important to note that clinical trials pertaining to these antigenic targets have yet to be initiated.

### Breast cancer

CAR T-cell therapy for breast cancer primarily targets specific members of the Receptor Tyrosine Kinase (RTK) family and certain proteins found on the surface of cells. Five prominent RTKs, namely HER2 [178, 179], EGFR [180, 181], c-mesenchymal-epithelial transition factor (c-MET) [182, 183], ROR1 [177] and AXL [184], have demonstrated satisfactory outcomes as targets for CAR-T cell therapy in preclinical models of breast cancer. A number of clinical trials studied these five RTKs, including HER2-CAR-T cell therapy (NCT04650451, NCT03740256, NCT03696030), ROR1-CAR-T cell therapy (NCT02706392), and cMET-CAR-T cell therapy (NCT03060356, NCT01837602).

Regarding surface proteins, despite extensive preclinical research on 11 surface proteins (MUC1, MSLN, CD70, CD44v6, CD133, EpCAM, CSPG4, ICAM-1, TEM8, TROP2, FRα) [40, 185–193], only 6 proteins have been further investigated in clinical trials as targets of CAR-T treatment, including MSLN (NCT02580747, NCT02792114, NCT02414269), MUC1 (NCT04020575, NCT04025216, NCT02587689), EpCAM (NCT02915445), CD70 (NCT02830724), CD133 (NCT02541370), and CD44v6 (NCT04427449).

In addition to RKTs and cytokines, other targets such as GD2, Natural Killer Group 2 (NKG2) ligands, and serum tumor markers are being explored. Corresponding clinical trials are ongoing (GD2: NCT03635632; NKG2D: NCT04107142; CEA: NCT02349724, NCT03682744, NCT04348643), with no results reported yet.

### Gastric cancer

Studies have indicated that antigens like HER2, CEA, MUC1, Claudin 18.2 (CLDN 18.2), EpCAM, B7H3, MSLN, and NKG2D serve as effective targets for CAR-T cell therapy in gastric cancer [194]. CLDN 18.2, which belongs to a protein group that controls the movement of molecules between cells by interacting with tight junctions, exhibits high expression levels in gastric and gastroesophageal junction adenocarcinomas [195]. Preclinical investigations have demonstrated the safe and effective targeting of CLDN18.2-CAR T cell therapy in CLDN18.2-positive patient-derived gastric cancer xenotransplantation models [196]. In Phase 1 trial NCT03874897, 37 patients with CLDN18.2-positive gastrointestinal cancer received three distinct doses of CAR-T cells targeting CLDN18.2. Notably, 94.6% of the patients experienced grade 1 or 2 CRS with no serious adverse effects; as for treatment efficacy, the objective response rate (ORR) reached 57.1% and the disease control rate (DCR) was 75%. Moreover, the survival rate at 6 months was 81.2% [159]. Other targets that entered clinical trials of gastric cancer primarily included HER2 (NCT04660929), CEA (NCT05396300), MUC1 (NCT05239143), EpCAM (NCT05028933, NCT03563326), MSLN (NCT03941626), EGFR (NCT03740256), B7H3 (NCT04864821) and NKG2DL (NCT04550663), with no conclusions reported yet.

### Pancreatic cancer

The targets extensively investigated in pancreatic cancer include MSLN, HER2, EGFR, CEA, and CD133, being particularly notable as a well-studied antigen in the field of immunotherapy for pancreatic ductal adenocarcinoma (PDAC). In Phase I clinical trial NCT01897415, 16 patients with refractory metastatic advanced PDAC were enrolled. Notably, there were no dose-limiting toxicities (DLT) or serious AEs reported, and 2 patients achieved a state of SD lasting 3.8 and 5.4 months, respectively [197]. Another Phase 1 clinical trial of CD133-targeting CAR T cell therapy (NCT02541370) [198] included 23 participants with solid tumors that had more than 50% CD133 expression. Among these participants, 7 had advanced pancreatic cancer and underwent 2 to 4 cycles of CAR-T cell therapy. The findings indicated that a patient with stage IV PDAC, who had numerous metastases prior to therapy, experienced a 40% decrease in tumor size after the initial cycle, and subsequent infusions of CAR-T cells resulted in a prolonged period of disease control for this individual. Overall, 3 patients achieved SD, 2 patients achieved PR, and the other 2 patients had progressive disease (PD). As for safety, the cohort exhibited grade 2 to 3 AEs, with 1 patient experiencing grade 4 leukopenia. In another Phase I study (NCT01869166), 16 patients with metastatic PDAC expressing EGFR levels exceeding 50% were enrolled, and they received EGFR-CAR-T cell therapy for 2 to 4 cycles [199]. Out of these patients, 58% encountered mild AEs such as fever, exhaustion, and nausea; 38% of the patients had lymphocytopenia in grades 3 and 4, and there were two cases of grade 3 pleural effusion and pulmonary interstitial exudation. In terms of efficacy, 8 patients achieved SD for 2–4 months, 4 patients realized PR for 2–4 months, 2 patients experienced PD, and the remaining 2 patients were lost to follow-up. A total of 11 patients including 2 individuals with PDAC expressed more than 50% HER2 participated in a Phase 1 HER2-CAR-T cells therapy trial (NCT01935843) [200] and received at least 2 cycles of HER2-CAR-T cell therapy. Although all patients experienced acute febrile syndrome, no instances of severe CRS were reported. Most AEs were reversible and treatable. Notably, two PDAC patients achieved SD, with durations of 5.3 and 8.3 months, respectively.

### Hepatocellular carcinoma (HCC)

GPC3, which is expressed in over 75% of patients with HCC but not in normal liver cells, is the target antigen most commonly utilized for CAR-T cell therapy in HCC [201]. Numerous studies have consistently shown the effectiveness of GPC3-CAR-T cells in suppressing the proliferation of xenografts derived from liver cancers. In a Phase 1 trial (NCT03198546) [202], an advanced HCC patient underwent intra-tumoral injection of GPC3-CAR T cells, leading to complete elimination of the tumor within a month. The only significant AE observed was severe fever.

CEA, a cell surface binding glycoprotein that is expressed in normal tissues at low levels while in a variety of solid tumor tissues at high levels, is another promising target antigen [203]. Steven et al. conducted a Phase 1b clinical trial (NCT02416466) [204]. 6 patients with CEA-positive liver metastases were administered CEA-CAR-T cells. The outcomes revealed remarkable tolerance without any occurrences of severe toxicity (grade 4 to 5), severe CRS, or neurotoxicity. The findings confirmed the overall well-tolerated characteristics of CEA-CAR-T therapy in liver cancer.

In a single-arm, open-label phase 2 clinical trial (NCT02541370), 21 patients diagnosed with advanced HCC received an infusion of CD133-CAR T cells [205]. The results revealed that 1 patient reached PR, stable disease was maintained for 2 to 16.3 months in 14 patients, and 6 patients experienced PD. Regarding safety, 4 patients experienced grade 3 hyperbilirubinemia, while 2 patients had grade 3 anemia. Additionally, there were no other severe AEs, thus confirming the efficacy and safety of CD133-CAR T cell therapy in advanced HCC patients.

Several other promising targets for CAR-T cell therapy in HCC include alpha-fetoprotein (AFP), B7H7, EGFRvIII, EPCAM, NKG2DL, MUC1, PD-L1, and CD147. Ongoing relevant clinical trials include: NCT03253289 (AFP), NCT05323201 (B7H3), NCT03941626 (EGFRvIII), NCT03013712 (EpCAM), NCT04550663 (NKG2DL), NCT04842812 (MUC1), NCT03672305 (PD-L1), NCT03993743 (CD147).

### Colorectal cancer (CRC)

The main targets for CAR T cell therapy in colorectal cancer include CEA [206], MSLN [207], Guanylyl Cyclase C (GUCY2C) [208], EpCAM [209], HER2 [210], and Doublecortin-like kinase 1(DCLK1) [211]. In a clinical trial NCT02349724 [212], 10 patients with relapsed and refractory metastatic colorectal cancer were administered CAR-T treatment with 6-level escalating dosage, and no severe AEs was observed. 2 individuals achieved SD for a duration exceeding 30 weeks, while 2 other patients experienced a reduction in tumor size. Another Phase I clinical trial (NCT02541370) [198] of CD133-CAR-T cell therapy enrolled 23 patients, including 2 individuals with colorectal cancer. Of the two colorectal cancer patients, both achieved SD. The primary AEs were anemia or thrombocytopenia (≤ grade 3).

Further targets currently under investigation in clinical trials encompass MUC1 (NCT05239143), HER2 (NCT04660929), MSLN (NCT05089266), NKG2DL (NCT04550663), EpCAM (NCT05028933), and GUCY2C (NCT05287165).

Comprehensive details of the clinical trials for the mentioned targets and other additional solid tumors were listed in Table 1.

## Conclusions

CAR-T cell therapy has made prominent advancements in the treatment of hematologic malignancies. Nevertheless, its efficacy in treating solid tumors is limited due to various factors, such as the lack of specific antigens, the complex TME, and the potential for treatment-related toxicity. This article delved into the difficulties linked to CAR-T cell therapy in solid tumors and explored a spectrum of strategies proposed by researchers. These strategies primarily revolve around pinpointing specific target antigens, designing multi-target or next-generation CAR-T cells to counter antigenic diversity, implementing engineering techniques to bolster T cell efficacy and mitigate T cell exhaustion. Moreover, creative solutions have been developed to address the limitations of T cell infiltration. Promising prospects are also presented by the combination of CAR-T cell therapy with other treatment methods like chemotherapy, radiotherapy, or immunotherapy. With extensive preclinical research and well-designed clinical trials, it is envisioned that CAR-T cell therapy will become a robust and promising therapeutic option for solid tumors in the foreseeable future.

## Data Availability

No datasets were generated or analysed during the current study.

## Abbreviations

CAR: Chimeric antigen receptor
ICIs: immune checkpoint inhibitors
PD-1: programmed cell death protein 1
PD-L1: programmed cell death 1 ligand 1
MHC: major histocompatibility complex
TME: tumor microenvironment
VH: variable heavy chain
VL: variable light chain
scFv: single chain fragment variable
ITAM: immunoreceptor tyrosine-based activation motif
FDA: the U.S. Food and Drug Administration
TSA: tumor-specific antigen
TAA: tumor-associated antigen
EGFR: epidermal growth factor receptor
HER2: human epidermal growth factor receptor 2
MSLN: mesothelin
HHLA2: human endogenous retrovirus-H long terminal repeat-associating protein 2
NSCLC: non-small cell lung cancer
FR: folate receptor
iCasp9: inducible caspase 9
Tregs: regulatory T cells
TAMs: tumor-associated macrophages
MDSCs: myeloid-derived suppressor cells
IDO: indoleamine 2, 3-dioxygenase
DCs: dendritic cells
IFNγ: interferon-γ
NECD: Notch receptor extracellular domain
NICD: Notch receptor intracellular domain
TNF: tumor necrosis factor
FA-TLR7-1A: Folate-targeted Toll-like receptor 7 agonist
ECM: extracellular matrix
MMP-9: matrix metalloproteinase-9
TR2: TNF-related apoptosis-inducing ligand-receptor 2
TGFβ: transforming growth factor beta
SwR: switch receptor
PSMA: prostate-specific membrane antigen
NK cells: natural killer cells
PI3K: phosphatidylinositol-3-kinase
shRNA: short-hairpin RNA
ST3GAL1: ST3 β -galactoside α -2,3-sialyltransferase 1
LFA-1: lymphocyte function-associated antigen-1
VEGF: vascular endothelial growth factor
ATCs: adoptively transferred T cells
HA: hyaluronic acid
CASCs: cancer-associated stromal cells
CAFs: cancer-associated fibroblasts
aSMA+: alpha smooth muscle actin-positive myofibroblasts
MSCs: mesenchymal stem cells
HSPG: heparan sulfate proteoglycan
HPSE: heparinase
FAP: fibroblast activation protein
DNMT3A: DNA methyltransferase 3 A
TCF7: T cell factor 7
LEF1: lymphoid enhancer-binding factor 1
ICOS: inducible T cell co-stimulator
AICD: activation-induced cell death
TLR7: Toll-like receptor 7
Runx3: Runt domain-related transcription factor 3
CTL: cytotoxic T lymphocyte
Trm: tissue-resident memory T cell
CRS: cytokine release syndrome
GM-CSF: granulocyte-macrophage colony-stimulating factor
GSDM: gasdermin
ASTCT: The American Society for Transplantation and Cellular Therapy
ICANS: immune effector cell-associated neurotoxicity syndrome
BBB: blood-brain barrier
CNS: central nervous system
CLL: chronic lymphocytic leukemia
ICD: immunogenic cell death
RT: radiotherapy
OVs: oncolytic viruses
OAV: oncolytic adenovirus
CCL5: chemokine (C-C motif) ligand5
CA9: carbonic anhydrase 9
MUC1: mucin 1
CEA: carcinoembryonic antigen
GBM: glioblastoma
ROR1: receptor tyrosine kinase-like orphan receptor 1
AE: adverse event
PR: partial response
SD: stable disease
mPFS: median progression-free survival
mOS: median overall survival
PSCA: prostate stem cell antigen
GPC3: Glypican-3
RTK: Receptor Tyrosine Kinase
c-MET: c-mesenchymal-epithelial transition factor
NKG2: Natural Killer Group 2
ORR: objective response rate
DCR: disease control rate
OS: overall survival
PDAC: pancreatic ductal adenocarcinoma
DLT: dose-limiting toxicities
PD: progressive disease
HCC: hepatocellular carcinoma
AFP: alpha-fetoprotein
GUCY2C: Guanylyl Cyclase C
DCLK1: doublecortin-like kinase 1
